# Impact of PM2.5 Exposure from Wood Combustion on Reproductive Health: Implications for Fertility, Ovarian Function, and Fetal Development

**DOI:** 10.3390/toxics13040238

**Published:** 2025-03-24

**Authors:** Paulo Salinas, Nikol Ponce, Mariano del Sol, Bélgica Vásquez

**Affiliations:** 1 Laboratory of Animal & Experimental Morphology, Institute of Biology, Faculty of Sciences, Pontificia Universidad Católica de Valparaíso, Valparaíso 2374631, Chile; 2 Doctoral Program in Morphological Sciences, Faculty of Medicine, Universidad de La Frontera, Temuco 4780000, Chile; nikol.ponce@ufrontera.cl (N.P.); mariano.delsol@ufrontera.cl (M.d.S.); 3 Center of Excellence in Morphological and Surgical Studies, Universidad de La Frontera, Temuco 4811230, Chile; belgica.vasquez@ufrontera.cl; 4 Department of Basic Sciences, Faculty of Medicine, Universidad de La Frontera, Avenida Francisco Salazar 01145, Temuco 4811230, Chile

**Keywords:** air pollution, reproduction, wood smoke, reproductive outcomes, ovary, PM2.5

## Abstract

This study evaluates the impact of PM2.5 exposure from wood combustion on reproductive health and fetal development using an experimental model in Sprague Dawley rats. The study was conducted in Temuco, Chile, where high levels of air pollution are primarily attributed to residential wood burning. A multigenerational exposure model was implemented using controlled exposure chambers with filtered (FA) and unfiltered (NFA) air. Second-generation (G2) female rats (*n* = 48) were exposed pregestationally (60 days) and gestationally (23 days) under four conditions: FA/FA, FA/NFA, NFA/FA, and NFA/NFA. PM2.5 concentration and composition were monitored using beta-ray attenuation and X-ray fluorescence spectrometry. Reproductive parameters, ovarian follicle counts, and hormonal levels were assessed via vaginal cytology, histological analysis, and chemiluminescence immunoassays. PM2.5 exposure disrupted estrous cyclicity (*p* = 0.0001), reduced antral and growing follicles (*p* = 0.0020; *p* = 0.0317), and increased post-implantation losses (*p* = 0.0149). Serum progesterone and estradiol levels were significantly altered (*p* < 0.05). Despite ovarian disruptions, fertility rates remained unchanged. These findings suggest that chronic exposure to wood smoke-derived PM2.5 adversely affects ovarian function and fetal growth without significantly impairing overall reproductive capacity. This study highlights the need for public health policies to mitigate wood smoke pollution.

## 1. Introduction

The frequent use of wood for residential heating, driven by cultural preferences and affordability, significantly contributes to air pollution. This practice emits not only fine particles (PM) and carbon monoxide (CO) but also carcinogens such as benzene and polycyclic aromatic hydrocarbons, posing substantial health risks [1,2,3,4]. In southern Chilean cities like Temuco, the predominant use of wood for residential heating generates severe pollution episodes that often exceed national regulatory standards, negatively impacting human health [5,6,7,8,9,10]. Temuco is identified as one of the most polluted cities in Latin America, with residential wood burning accounting for 90% of fine particle emissions [11,12].

Most studies on air pollution and PM2.5 have focused on particles generated by petroleum combustion, while those from wood burning have received considerably less attention. This is particularly concerning in regions where wood is a significant source of domestic energy and substantially contributes to the total PM2.5 burden in the air. These particles not only affect the cardiovascular and respiratory systems but also act as endocrine disruptors, with potential implications for female fertility and fetal development. In vitro and in vivo studies indicate that wood smoke pollutants can induce oxidative stress, genotoxic effects, and endocrine alterations, including estrogenic, anti-estrogenic, and anti-androgenic effects, as well as interference with the thyroid axis. These mechanisms can exacerbate metabolic disorders such as insulin resistance and obesity, conditions closely associated with infertility [13,14,15,16].

While the relationship between air pollution and adverse reproductive and fetal outcomes is well documented [17,18], most experimental studies have focused on PM2.5 derived from petroleum combustion, particularly regarding its effects during pregnancy. However, occupational and epidemiological research suggests that maternal exposure prior to conception may also have significant implications [18,19,20,21,22]. Despite the extensive evidence on the health effects of PM2.5 from fossil fuel sources, there is a notable lack of studies specifically addressing the impact of PM2.5 generated by wood combustion on reproductive health and fetal development. This knowledge gap is particularly relevant in regions where wood burning is the primary source of air pollution. Recent evidence [23] suggest that that pregestational and gestational exposure to PM2.5, primarily sourced from wood combustion for home heating, significantly affects placental vascular morphophysiology and fetal size. Their findings highlight alterations in placental angiogenesis and oxygen diffusion capacity, accompanied by decreased fetal weight and crown–rump length, as well as increased HIF-1α expression. These results underscore the dual impact of PM2.5 exposure during pregestational and gestational stages, demonstrating that placental adaptations to hypoxia may compromise fetal growth and oxygen supply. Additionally, prenatal exposure to air pollution from wildfires has been linked to birth defects [24], and similar pollution exposure has been associated with epigenetic changes in the placenta, suggesting a link between air pollution exposure during pregnancy and long-term health effects [25].

Based on this context, we hypothesize that pregestational and gestational exposure to PM2.5, primarily originating from residential wood combustion smoke, affects reproductive organs and outcomes, including fetal development. This study aims to explore the effects of wood smoke on maternal and fetal reproductive, hormonal, and gestational indicators, focusing on pregestational and gestational exposure in residential environments. This study aspires to fill the gap in the literature on the effects of PM2.5 derived from wood burning and to provide a more comprehensive understanding of the potential risks associated with this source of pollution.

## 2. Materials and Methods

### 2.1. Location and Air Pollution Exposure

This study was conducted in Temuco, La Araucanía Region, Chile (38°44′59.4″ S, 72°37′07.8″ W), in an area characterized by 24 h residential wood burning as the primary source of heating. No industrial pollution sources were identified in the region. The association between PM2.5 exposure from wood smoke and reproductive, maternal, and fetal parameters in rats was evaluated using a time-stratified crossover case design. This approach simulated a gradient in particle levels by employing adjacent filtered- and unfiltered-air chambers, following the method described previously [26]. This study was carried out during the austral winter, from 15 June to 30 September 2021.

### 2.2. Chambers and Filtration System

**Figure 1:**
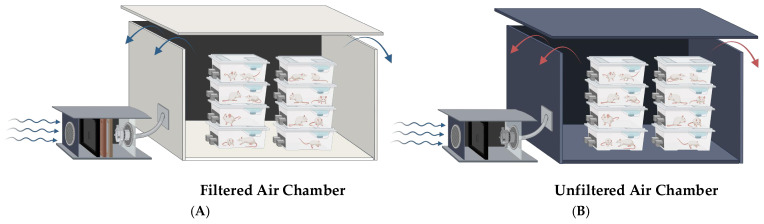
Experimental exposure chambers. (**A**) Filtered-air (FA) chamber: a controlled environment designed to provide filtered air, effectively removing PM2.5 and other airborne pollutants. (**B**) Unfiltered-air (NFA) chamber: an exposure chamber that allows subjects to inhale unfiltered ambient air, simulating real-world conditions of PM2.5 pollution. FA = filtered air; NFA = non-filtered air (unfiltered air). The red and blue arrows indicate the ambient air escaping from the exposure chamber to the external environment.

The exposure chambers consisted of two identical structures (2.1 m × 2.0 m × 2.1 m; Figure 1) positioned side by side, with air introduced at a constant flow rate of 20 m^3^/min. One chamber filtered the air using a three-stage system: metallic filters for large particles, HEPA PH97 filters that removed 99.97% of particles larger than 0.3 microns, and a Purafil PSA 102 gas filtration system with Purafil Select filter media (Purafil, Inc., Doraville, GA, USA). The other chamber received unfiltered air. Both chambers maintained consistent environmental conditions for temperature and humidity, which were regularly verified using calibrated sensors placed at strategic points within the structures. Air distribution was ensured to be uniform by a fan (150 m^3^/h) and a wide upper exhaust outlet.

### 2.3. Air Analysis and Composition of PM2.5

Particulate matter and gaseous pollutant concentrations were monitored through multiple complementary analytical approaches. PM2.5 concentrations underwent continuous 24 h monitoring in both experimental chambers using a digital analyzer, while ambient outdoor concentrations were measured via beta-ray attenuation monitoring [27] using a BAM 1020 Particle Monitor (Met One Instruments, Inc., Grant Pass, OR, USA). This instrument, featuring a Beta Detector Type Photomultiplier tube and organic plastic scintillator, operated at a 16.7 L/min flow rate, with results expressed in µg/m^3^. Gaseous pollutant monitoring included ambient CO measurements as 8 h moving averages using Ultraviolet Absorption Photometry (Teledyne T300, San Diego, CA, USA), while NO_2_ concentrations were determined as 24 h averages through Gas-Phase Chemiluminescence using a Thermo 42i instrument (Waltham, MA, USA). Sample collection was performed by Algoritmos y Mediciones Ambientales SpA (Temuco, Chile) at the Las Encinas Monitoring Station of the National Air Quality Information System, situated 200 m from the exposure chamber site. Data access was facilitated through the National Air Quality Information System website. Notably, the filtering system did not retain pollutant gasses, resulting in comparable NO_2_ and CO concentrations between chambers. Comprehensive PM2.5 compositional analysis employed multiple analytical techniques. Elemental analysis (Na, Al, Si, P, S, K, Ca, Ti, V, Fe, Ni, Cu, Zn, and Pb) was conducted using scanning electron microscopy coupled with energy-dispersive X-ray analysis (EDX). Additional non-destructive elemental characterization utilized a portable TRACER 5 X-ray fluorescence spectrometer, operating on energy-dispersive X-ray fluorescence principles. Metal detection was accomplished using a Dual LIBS-Raman Spectroscopic Microscope (Unchained Labs, Pleasanton, CA, USA).

### 2.4. Animals

A total of 48 second-generation (G2) Sprague Dawley rats were used, housed in the university animal facility under controlled conditions (18–26 °C, 40–60% humidity) and fed a balanced diet ad libitum. The selection of second-generation rats was justified to eliminate potential confounding effects associated with pregestational exposure in previous generations, ensuring that the observed responses could be attributed exclusively to the experimental conditions. Additionally, to minimize paternal bias, only males raised in controlled animal facility conditions were included. All procedures complied with NIH guidelines [28] and were approved by the Bioethics Committee of Universidad de La Frontera (Approval No. 122/2020).

### 2.5. Experimental Design and Groups

**Figure 2:**
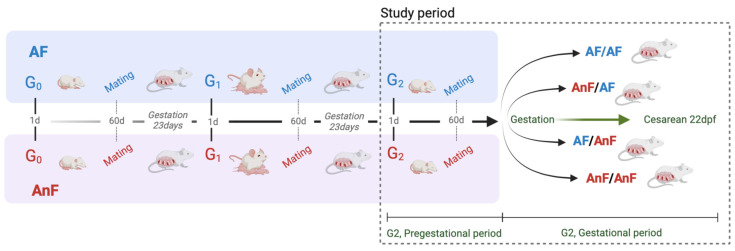
G2 rat breeding design. A diagram illustrating the breeding and developmental progression from the initial generation (G0) to the second generation (G2). It details the mating stages, gestation periods, and different environmental conditions (FA and NFA) to which the rats were exposed. The study period and methodological framework used to assess reproductive and developmental parameters in G2 rats are also included. FA = filtered air; NFA = non-filtered air (unfiltered air).

This study aimed to evaluate the impact of PM2.5 exposure from wood smoke on reproductive health, with a specific focus on estrous cyclicity, ovarian morphology, and gestational outcomes. To achieve this, a controlled exposure model was implemented using filtered-air (FA) and non-filtered-air (NFA) chambers, allowing for the assessment of chronic exposure effects in an environmentally relevant setting. The experimental design encompassed a multigenerational exposure study examining wood smoke pollution effects across three generations of rats, with particular emphasis on G2 to assess pregestational and gestational impacts (Figure 2). The study commenced with G0 breeding (10 males and 10 females) in filtered-air (FA) or non-filtered-air (NFA) chambers. G1 offspring remained in their respective environments, subsequently producing G2 rats through intra-generation mating. The G2 experimental protocol spanned pregestational (60 days) and gestational (23 days) periods, with subjects distributed across four treatment groups: FA/FA (control group, *n* = 12, representing G2 females experiencing both development and pregnancy in filtered air), FA/NFA (*n* = 12, development in filtered air followed by pregnancy in unfiltered air), NFA/FA (*n* = 12, development in unfiltered air followed by pregnancy in filtered air), and NFA/NFA (*n* = 12, both development and pregnancy in unfiltered air). Study participation required rats to meet specific inclusion criteria: exclusive birth and development in designated environments (FA or NFA), the demonstration of good general health, and regular estrous cycles confirmed through vaginal cytology. Exclusion parameters encompassed preexisting disease indicators, irregular estrous cycles, and body weight anomalies relative to established Sprague Dawley rat standards under experimental conditions. Physiological monitoring included the regular assessment of vaginal cytology, tridaily body weight measurements, and gestational blood pressure monitoring following established protocols [29].

### 2.6. Blood Collection and Hormone Measurements

Blood samples were collected from pregnant G2 rats 21 days post-fertilization (dpf) via lateral tail vein puncture [30]. Plasma was extracted from heparinized tubes after centrifugation and stored at −20 °C until analysis. Serum progesterone and estradiol levels were measured using MAGLUMI^®^ Prg and Estradiol assays (Snibe Co., Ltd., Shenzhen, China), with ranges of 0.13–80 ng/mL and 8–6000 pg/mL, respectively. Both assays had intra-assay CVs < 8% and inter-assay CVs < 9%. Values below detection limits were recorded as half the limit, with appropriate controls and standardization. Hormone quantification was performed via chemiluminescence immunoassay using the MAGLUMI 600 analyzer (Snibe Co., Ltd., Shenzhen, China).

### 2.7. Maternal, Fetal, and Reproductive Indices

Ovarian cyclicity. The estrous cycle of 12 G2 rats housed in FA and NFA chambers was analyzed through daily vaginal lavages and cytology starting at six weeks of age. The phases of the cycle were determined based on the relative proportion of cornified epithelial cells, nucleated epithelial cells, and leukocytes in the samples [31,32]. Cyclicity was defined as the number of proestrus-to-estrus events and the total days in estrus during the monitoring period, evaluated by two blinded observers.

Counting of ovarian follicles. The number of ovarian follicles was estimated in both ovaries (left and right) from six females per group of G2 rats in estrus [33]. Ovaries were fixed in 4% paraformaldehyde, dehydrated in graded ethanol, and sectioned histologically at 20 µm intervals. Sections were stained with hematoxylin and eosin and digitized for analysis. Follicles were classified into four categories, small (primordial and primary), growing, antral, and preovulatory, based on their morphology and cellular characteristics. All measurements were performed by two trained observers blinded to the group assignments.

Evaluation of reproductive capacity. This corresponds to the functional capacity of the reproductive system to achieve pregnancy and result in a successful birth. Reproductive parameters were evaluated in G2 rats, including the mating rate ([females with vaginal plugs/total cohoused females] × 100), mating time (time from cohousing to the detection of a vaginal plug or spermatozoa), fertility rate ([pregnant females/mated females] × 100), and pregnancy rate ([females with live offspring/pregnant females] × 100).

Gestational outcomes. At 21 dpf, six pregnant rats per group underwent ovariohysterectomy, while the remaining six continued to full term. Uteri were inspected to count fetal vesicles (live and dead), measure the crown–rump length, and record the fetal weight. The corpora lutea in both ovaries were quantified. Indices were calculated following US EPA guidelines [34], including the implantation index ([implantation sites/corpora lutea] × 100), pre-implantation losses (proportion of fertilized oocytes that failed to implant in the endometrium: [corpora lutea − implantation sites]/corpora lutea × 100), and post-implantation losses (proportion of implanted embryos that failed to develop to full term: [implantation sites − full-term offspring]/implantation sites × 100).

### 2.8. Statistical Analysis

To test our hypothesis that pregestational and gestational exposure to PM2.5 affects reproductive organs and outcomes, including fetal development, we employed a combination of parametric and multivariate statistical methods. Continuous variable normality was assessed using the D’Agostino–Pearson test, as normality is a prerequisite for applying parametric tests such as one-way ANOVA and Student’s *t*-test. These tests were selected because they allowed us to compare differences in ovarian follicle counts, gestational outcomes, and reproductive capacity between exposed and control groups. Ensuring normality minimized bias by preventing the inappropriate use of parametric methods. To identify pollution sources, principal component analysis (PCA) was employed as a dimensionality reduction technique, summarizing correlated environmental variables into uncorrelated components while preserving the maximum variance. A normalized varimax rotation was applied to maximize the variance distribution among factor loadings, enhancing interpretability and allowing singular factor assignment where possible. Factor retention followed the Kaiser criterion (eigenvalues > 1.0), ensuring that only components explaining meaningful variance were included in the analysis. Factor loadings were interpreted using standard cut-offs: values below 0.32 were considered weak, while those above 0.71 were strong indicators of component membership. To analyze the effects of PM2.5 exposure on gestational and reproductive outcomes, we used one-way ANOVA with Holm–Šídák multiple-comparisons tests. The Holm–Šídák method was chosen over the Bonferroni correction due to its ability to control for type I error while maintaining statistical power, thereby reducing the risk of false negatives in multiple-group comparisons. Differences in reproductive capacity between groups were evaluated using Student’s *t*-test, as this test provides an appropriate comparison for independent samples when normality assumptions are met. Statistical significance was set at *p* < 0.05 with 95% confidence intervals. The assessment of G1 rat gestational growth trajectories was performed to evaluate potential effects of chronic PM2.5 exposure on fetal development, given that fetal weight is a key indicator of the intrauterine environment and gestational health. A generalized linear mixed model (GLMM) was implemented in R Studio (RStudio2023.09.1) using the “mgcv” [35], “lme4” [36], “lmerTest” [37], and “pbkrtests” [38] packages. The model structure incorporated weight as the response variable, group designation as a fixed factor, and temporal measurements as well as individual replicates as random factors to account for within-subject variability and the repeated-measures structure. This model was chosen due to the longitudinal nature of the data, where repeated weight measurements within the same individuals introduced correlations that had to be accounted for to avoid biased estimates. To minimize bias, data collection and analysis were conducted by blinded observers, and sample selection was randomized within experimental groups to prevent selection bias.

## 3. Results

### 3.1. Climatic Conditions at the Study Site

According to the Chilean Meteorological Directorate (DM, 2024), the average temperature in Temuco during the study period was 7.9 °C, with an 88% relative humidity, wind speeds below 6 m/s, 94.55 mm of precipitation, and an average of 19 overcast days per month.

### 3.2. Air Pollution Exposure

**Figure 3:**
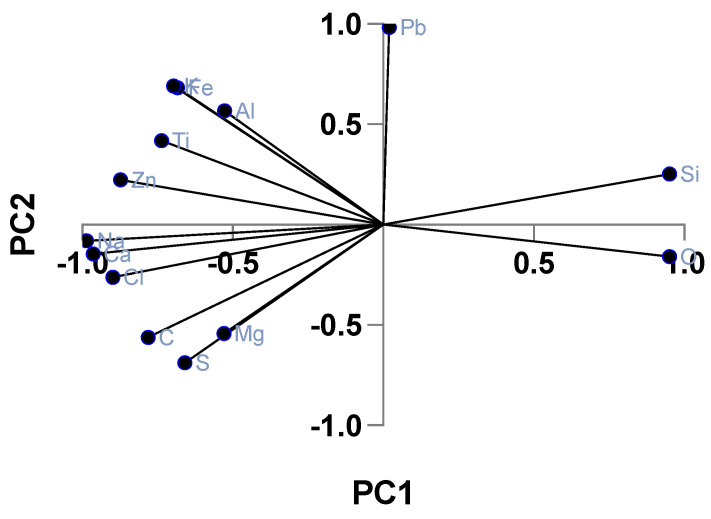
A principal component analysis (PCA) biplot with varimax rotation of the elemental composition of PM2.5. The biplot represents the distribution of elements in PM2.5 along the first two principal components (PC1 and PC2) after varimax rotation, which optimizes factor loadings for better interpretability. The direction and length of each vector indicate the strength and contribution of each element to the respective principal component. Elements such as Pb and Si exhibit strong positive loadings on PC1, suggesting that they originate from similar sources, possibly industrial emissions or geological materials. Conversely, Na, Cl, and Ca align along PC2, likely associated with sea-salt aerosols or soil-derived particles. Elements like Mg and S are more evenly distributed across both components, implying a contribution from mixed sources, such as biomass combustion and secondary aerosol formation. This analysis helps distinguish potential sources of particulate matter, supporting the identification of pollution origins relevant to exposure assessments in the study.

The daily average concentrations of pollutants at the exposure site were 48.8 µg/m^3^ (±36.1; CV = 74%) for PM2.5, 56.9 mg/m^3^ (±38.3; CV = 67.3%) for PM10, and 0.78 ppm (±0.49; CV = 61.5%) for CO (Table 1). Within the NFA chamber, PM2.5 concentrations averaged 44.6 µg/m^3^ (±9.8; CV = 21.9%), closely matching ambient levels, while in the FA chamber, the average PM2.5 concentration was significantly reduced to 3.0 µg/m^3^ (±1.3; CV = 34.3%; *p* < 0.001), achieving a 94% reduction. Maternal exposure during gestation was estimated by calculating the total inhaled air volume (1299 L over 22 days at an inhalation rate of 0.041 l/min) and multiplying it by the average PM2.5 concentration. For NFA/NFA females, the cumulative inhaled PM2.5 during gestation was 57,934 µg/m^3^, equivalent to 2633.40 µg/m^3^ daily. In contrast, FA/FA females were exposed to significantly lower values of 3896.97 µg/m^3^ cumulatively and 177.14 µg/m^3^ daily. The elemental composition of PM2.5, analyzed via EDX, is detailed in Table 2, while Table 3 presents PCA results, identifying two principal components. Figure 3 illustrates the principal component analysis (PCA) with normalized varimax rotation, displaying the factor loadings of each element on the two main principal components (PC1 and PC2). Higher absolute loadings indicate a stronger contribution of an element to a given component. Elements such as Pb and Si exhibit strong associations with PC1, suggesting a likely geochemical or industrial source. In contrast, Mg and S show more balanced distributions across both PC1 and PC2, indicating multiple or mixed sources. This factor distribution helps infer the relative influence of different emission sources on PM2.5 composition

### 3.3. Blood Collection and Measurement of Serum Hormone Levels

**Figure 4:**
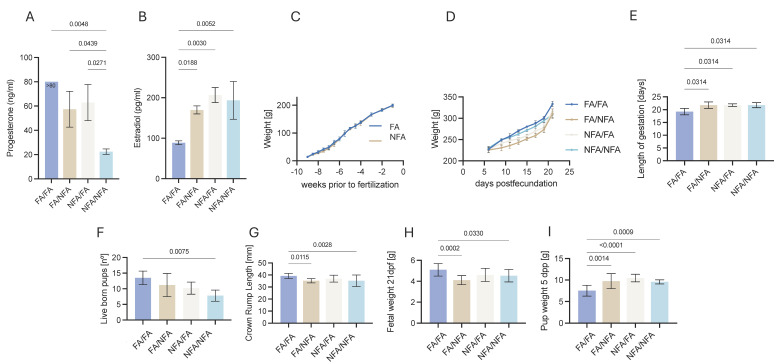
Impact of PM2.5 exposure on reproductive hormones, maternal weight, and fetal development in G2 rats and their G3 offspring. In G2 rats at 21 days post-fecundation (21 dpf): (**A**) progesterone serum hormone levels, (**B**) estradiol serum hormone levels, (**C**), weight during pre-pregnancy period, (**D**) weight during gestational period, and (**E**) gestational length (days). In G3 fetuses: (**F**) live fetuses per litter, (**G**) crown–rump length, (**H**) fetal weights at 21 dpf, (**I**) fetal weights at 5 days post-natal.

The serum hormone levels of G2 rats are presented in Figure 4. The AF/AF group exhibited significantly higher serum progesterone concentrations (Figure 4A) and lower estradiol levels (Figure 4B) compared to all other groups (*p* < 0.05). In contrast, G2 rats exposed to NFA during the pregestational and/or gestational periods showed significantly increased estradiol levels. Notably, estradiol concentrations in the FA/NFA group were significantly lower than in the NFA/FA group (*p* < 0.05), as reflected in the non-overlapping confidence intervals in Figure 4B. These findings suggest that exposure timing may have differentially influenced estradiol regulation, with pregestational exposure exhibiting a distinct effect compared to gestational exposure alone.

### 3.4. Maternal, Fetal, Reproductive, and Gestational Indices

Based on our data, maternal weight in the reference group (FA/FA) ranged from 213.7 g to 352.0 g throughout the gestational period, with an average weight gain of 118.3 g between day 6 and day 21 post-fertilization (DPF). In contrast, rats exposed to NFA (pregestationally and/or gestationally) exhibited lower weight gain compared to the control group, suggesting an adverse impact of environmental exposure on gestational weight progression. Maternal body weight trajectories were analyzed during pregestational and gestational periods (Figure 4C,D). During pregestation, both groups demonstrated weight increases approaching fertilization, with the FA group showing a marginally steeper trajectory, though without statistical significance. Gestational weight patterns revealed FA/FA subjects exhibiting maximal gains, while NFA/NFA subjects showed minimal gains, with mixed-exposure groups (FA/NFA, NFA/FA) displaying intermediate patterns more closely aligned with FA/FA outcomes. The FA/FA and NFA/NFA comparison during gestation approached but did not achieve significance (*p* = 0.0764). Multivariate analysis of gestational growth curves (Figure 4D) identified significant distinctions between consistent-environment groups (FA/FA, NFA/NFA) versus mixed-condition groups (FA/NFA, NFA/FA). Table 4 presents the fixed-effects analysis of a mixed linear model evaluating the differences in weight between groups. The intercept represents the estimated average weight for the FA/FA reference group, while the coefficients for the other groups indicate their respective deviations from this baseline. A negative estimate signifies a lower weight compared to the FA/FA group. The standard error (Std. Error) reflects the variability of the estimate, while the *t*-value represents the magnitude of the difference in relation to its standard error. The *p*-values (Pr(>|t|)) indicate the statistical significance of these differences, with lower values suggesting stronger evidence against the null hypothesis. In this analysis, all groups exposed to NFA (FA/NFA, NFA/FA, and NFA/NFA) exhibited significantly lower weight compared to the AF/AF group, as indicated by the negative estimates and highly significant *p*-values. The most pronounced difference was observed in the FA/NFA group (*p* = 3.35 × 10^−14^), followed by NFA/FA (*p* = 7.13 × 10^−7^) and NFA/NFA (*p* = 0.0164). This suggests that exposure to NFA, particularly in the pregestational phase, is associated with a significant reduction in weight. Table 5 presents the pairwise weight contrasts between experimental groups, evaluated using a mixed linear model with Kenward–Roger degrees-of-freedom adjustment and Tukey’s correction for multiple comparisons. Each contrast shows the estimated difference in weight between two groups, where positive values indicate that the first group in the comparison had a higher average weight than the second, while negative values indicate the opposite. The standard error reflects the variability of each estimate, and the *t*-ratio quantifies the strength of the observed differences. The most pronounced difference was observed between FA/FA and FA/NFA (23.61 g; *p* < 0.0001), followed by FA/FA and NFA/FA (15.14 g; *p* < 0.0001), suggesting a significant impact of NFA exposure on weight reduction. Additionally, FA/NFA rats weighed significantly less than NFA/NFA rats (−16.90 g; *p* < 0.0001), further supporting the role of pregestational exposure in modulating body weight.

### 3.5. Fetal Index

Significant differences were observed in the number of live G3 fetuses per litter across groups (Figure 4F). The crown–rump length (CRL) at 21 dpf was significantly greater in the FA/FA group (39.2 ± 2.11 mm) compared to the NFA/NFA group (35.2 ± 4.75 mm), reflecting a 12% increase (*p* < 0.0001; Figure 4G). Similarly, the fetal weight at 21 dpf was 13% higher in the FA/FA group (5.09 ± 0.59 g) compared to the NFA/NFA group (4.53 ± 0.59 g; *p* < 0.0001; Figure 4H). However, the post-natal fetal weight at 5 days was significantly lower in the FA/FA group (7.52 ± 1.26 g) compared to the NFA/NFA group (9.58 ± 0.47 g), representing a 22% reduction (*p* < 0.0001; Figure 4I).

### 3.6. Reproductive Index

Key reproductive parameters, including ovarian cyclicity and estrous cycle characteristics, are detailed in Table 6. PM2.5 exposure significantly impacted the duration of the estrous cycle (*p* = 0.0001) and estrus (*p* = 0.0057), leading to a reduction in the number of observed cycles (*p* = 0.0432). Furthermore, PM2.5 exposure was associated with a significant decrease in the number of growing (*p* = 0.0317) and antral follicles (*p* = 0.0020) in NFA rats. Indicators of reproductive capacity, such as mating (*p* = 0.1456), fertility (*p* = 0.540), and pregnancy rates, showed no significant differences between groups.

### 3.7. Gestational Index

Gestational outcomes across the four study groups are summarized in Table 7. PM2.5 exposure had a significant impact on the average rate of live-born fetuses per litter (*p* = 0.0125) and implantation rate (*p* = 0.0149), with a decrease of at least 40% in the NFA/NFA group. This reduction appeared to be influenced by both pregestational and gestational exposure. However, no significant differences were observed between groups in the number of live fetuses per litter (*p* = 0.3485), pre-implantation losses (*p* = 0.1323), or post-implantation losses (*p* = 0.3203).

## 4. Discussion

This study, conducted in an area where wood burning for domestic heating is the primary source of PM2.5 emissions, provides a detailed perspective on how these particles affect reproductive, fetal, and gestational parameters. For the first time, the effects of maternal exposure to wood smoke, both before and during gestation, were evaluated in two successive generations of rats (G1 and G2). The results demonstrated alterations in the estrous cycle, a reduction in antral and growing follicles, and a decrease in litter size and implantation rates in exposed rats. However, it is important to note that parameters related to the reproductive capacity of mating pairs, such as fertility and pregnancy rates, were not significantly affected, suggesting the presence of potential compensatory mechanisms in response to atmospheric pollutants.

The climatic conditions in Temuco during the study period favored the accumulation and persistence of pollutants due to high humidity, low wind speeds, and cold temperatures. These meteorological factors, combined with the widespread use of wood stoves for residential heating, contributed to an average PM2.5 concentration of 48.8 ± 36.1 μg/m^3^ (CV: 74.0%) during the study period, significantly exceeding national regulatory standards (MMA, 2024). On an annual scale, PM2.5 concentrations averaged 26.2 ± 30.6 μg/m^3^ (CV: 117%), highlighting the pronounced seasonal variability in pollution levels. Compared to Santiago, where wood burning is less frequent, PM2.5 concentrations in Temuco are markedly higher, reinforcing the notion that wood combustion is the predominant source of air pollution in the region [1,4,8,39]. Previous studies have established that approximately 90% of PM emissions in Temuco originate from residential wood burning, a practice sustained by its low-cost relative to other heating alternatives [4,40]. The strong seasonal dependence of these emissions results in critical pollution episodes during winter, leading to substantial daily fluctuations in PM2.5 levels, as reflected by the high coefficients of variation observed. These fluctuations are further influenced by meteorological and topographical factors that limit pollutant dispersion. Temuco is situated in a valley, and the combination of low wind speeds and frequent temperature inversions during colder months facilitates the trapping and accumulation of PM2.5 near the ground, exacerbating air pollution levels. As a result, PM2.5 concentrations can vary significantly from day to day, driven primarily by changes in heating activity and atmospheric conditions rather than by multiple emission sources.

Given these environmental characteristics, the air used in the exposure chambers closely reflects real-world pollution conditions in Temuco. The high PM2.5 concentrations observed in the chamber and the predominant elemental composition characteristic of wood smoke [1,2,3,4] make it an adequate model for studying the health effects of chronic exposure to wood smoke-derived particulate matter. However, it is important to recognize that individual exposure levels among Temuco’s residents may vary, depending on factors such as proximity to emission sources, ventilation conditions, and time spent indoors versus outdoors.

### 4.1. On Exposure and PM2.5

The PM2.5 concentrations recorded in ambient air and the unfiltered-air chamber during the exposure period significantly exceeded the WHO’s safety threshold of 10 µg/m^3^ annually [41] (41 WHO, 2021). Although lower than previously reported values—175 µg/m^3^ [42], 135.4 µg/m^3^ [4], and 54.5 µg/m^3^ [43]—these differences likely stemmed from variations in climatic conditions and emission source characteristics across regions. The considerable variability observed in PM2.5 levels, reflected in the high coefficients of variation (74% during the study period, 117% annually) and standard deviations, indicates substantial day-to-day and seasonal fluctuations in pollution levels. Given that residential wood burning is the primary emission source in Temuco, this variability is likely driven by changes in heating activity and meteorological conditions, such as temperature inversions and low wind speeds, which trap pollutants and affect their dispersion and accumulation in the urban environment. These findings are consistent with previous studies [4,40], who documented substantial spatial and temporal fluctuations in PM2.5 concentrations. Mean levels of 135.4 µg/m³ have been reported [4], while a median of 44.4 µg/m³ associated with indoor wood burning has been documented [40]. The present study’s average daily concentration of 48.8 µg/m^3^ reflects moderate levels relative to these reports, with observed differences attributable to the study period, emission source characteristics, prevailing climatic conditions, and air quality control measures. Despite these variations, the findings provide critical insights into air quality trends in Temuco, reaffirming the dominant role of residential wood combustion as a primary PM2.5 source. These data not only contextualize the exposure conditions examined but also contribute to a broader understanding of the cumulative impact of wood burning on air quality and public health in the region.

This study employed principal component analysis (PCA) to characterize the elemental composition of PM2.5 in Temuco, a city burdened by severe air pollution primarily resulting from residential wood combustion. The rotated component plot (Figure 3) and the rotated component matrix (Table 3) illustrate the principal component analysis (PCA) results, identifying the main patterns in PM2.5 elemental composition during the study period. The first two principal components (PC1 and PC2) together explain 88.31% of the total variance, meaning that they capture the majority of the variability in the dataset. PC1 represents the dominant pattern in elemental distribution, grouping elements with similar emission sources or physicochemical behavior, while PC2 differentiates secondary variations among elements. The significant correlation between these components (r = 0.8; *p* < 0.05) suggests that certain elements exhibit shared environmental influences, such as atmospheric dispersion or combustion-related processes. This analysis helps distinguish major contributors to PM2.5 composition, allowing for a better understanding of the exposure conditions in Temuco.

Through varimax rotation, PCA identifies distinct elemental groupings, with the first component encompassing C, Na, Cl, Ca, Mg, and S, while the second comprises Al, K, Fe, Zn, Ti, and Pb. These distributions align with wood combustion emissions, a major pollution source in firewood-dependent regions [44,45]. The presence of carbon, likely in the form of polycyclic aromatic hydrocarbons (PAHs), corroborates previous findings in Temuco [46], while soluble potassium (Ksol) serves as a recognized marker for wood smoke-derived PM2.5 [10,45]. The compositional complexity of PM2.5 reflects both primary and secondary pollutant interactions, with secondary pollutants and soil-derived elements, such as Al, Na, Cl, Ca, and Mg, frequently combining with smoke emissions [44]. Industrial metals, including Fe, Zn, and Pb, show indirect incorporation patterns, while sulfur, commonly present as sulfate from SO2 emissions during combustion [47], appears consistently across samples.

The presence of heavy metals such as Pb and Zn in PM2.5 is of particular concern due to their established associations with reproductive health risks, including reduced fertility [48], hormonal dysregulation [49], and implantation difficulties [50]. These findings underscore PM2.5’s complex composition, which integrates direct emissions and secondary formation processes. The variability in PM2.5 composition, influenced by wood type, combustion temperature, and efficiency [51], highlights the necessity of targeted pollution control strategies. Although this study did not assess organic pollutants such as PAHs, prior research has identified their role as significant risk factors [46]. While the mechanisms by which air pollution affects reproductive function remain unclear, particularly regarding ovarian cyclicity, estrous cycle duration, estrus length, and follicular development, the results suggest that PM2.5 components may impair reproductive health through endocrine disruption or direct tissue damage.

### 4.2. Endocrine Results in G2 Mother Rats

The results revealed differences in plasma concentrations of progesterone and estradiol between pregnant females exposed and unexposed to PM2.5 on gestational day 21 (dpf). Early prenatal exposure to PM2.5 is associated with increased concentrations of pregnenolone and maternal androgens during the late stages of gestation [52]. Previous studies suggests that PM2.5 acts as an endocrine disruptor, altering normal hormonal function, which may modify the production and regulation of reproductive hormones such as progesterone and estradiol [14]. Pregnenolone, a key precursor in steroid synthesis, influences androgen and estradiol production. Elevated pregnenolone levels could enhance estradiol synthesis, which may explain the increased concentrations of this hormone in females exposed to PM2.5. Furthermore, PM2.5 exposure could disrupt enzymatic activity involved in steroid biosynthesis [52], leading to an imbalance in hormone production. Toxic components of PM2.5, such as polycyclic aromatic hydrocarbons (PAHs), may induce or inhibit enzymes responsible for converting pregnenolone into progesterone and estradiol, thereby contributing to the observed hormonal alterations. These findings suggest that changes in progesterone and estradiol levels in females exposed to PM2.5 may result from complex interactions between pollutants, hormonal disruption, and enzymatic effects. Such interactions likely affect hormone synthesis and regulation, explaining the patterns observed in this study.

### 4.3. On the Body Weight of G2 Mother Rats Before and During Gestation

The body weight of G2 mother rats before and during gestation was evaluated using two generalized linear mixed models. In the first model, the factors time and replication were included as random effects, but the low variability attributed to replication justified their exclusion in the second model. The latter revealed significant weight differences among the various air exposure regimens (Table 4). Rats exposed to combinations of filtered and unfiltered air exhibited average weight reductions of 23.615 g, 15.143 g, and 6.714 g, depending on the exposure period. Continuous exposure to filtered air appeared to be associated with better health and growth conditions, whereas rats exclusively exposed to unfiltered air experienced less pronounced effects compared to those subjected to mixed regimens. The lack of statistical significance in certain comparisons following Tukey’s adjustment (Table 5) suggests that the magnitude of the effect might be small, necessitating a larger sample size to detect significant differences. These findings highlight the critical role of air quality during gestation, as exposure to unfiltered air during this period was associated with the greatest weight differences. This outcome may be related to increased fetal developmental vulnerability or disruptions in placental function [53]. However, post hoc adjustments to control for Type I errors reduce statistical power, which is a potential limitation in detecting subtle effects across the different exposure regimens.

### 4.4. On the Fetuses

The reduction in litter size observed in females exclusively exposed to unfiltered air during pregestational and gestational stages can be attributed to the adverse effects of prolonged PM2.5 exposure. This exposure has been associated with negative reproductive outcomes through mechanisms such as oxidative stress, endocrine disruption, and alterations in placental function [54,55,56]. Maternal exposure to PM2.5 during gestation was also linked to decreases in crown–rump length (CRL) and fetal weight, consistent with previous studies on fossil fuel emissions [57,58]. Similar research has connected gestational exposure to reductions in fetal growth in specific stages, such as E18.5 [59], as well as alterations in placental autophagy and metabolic function, affecting fetal development and post-natal programming [53,60]. However, uncertainty remains regarding the critical period of PM2.5 exposure and its impact on fetal growth parameters [53,61]. The post-natal weight gain observed in offspring exposed exclusively to unfiltered air during the first 5 days after birth—exceeding even that of offspring from mothers exposed solely to filtered air—may be explained by mechanisms such as post-natal compensatory growth, differences in lactation or maternal behavior, metabolic regulation influenced by environmental factors [62,63,64,65], or increased resilience developed in response to stress factors [66] (Pryor et al. 2022). While these factors offer plausible explanations, it is essential to avoid interpreting the observed weight gain as indicative of better health without a comprehensive analysis of the overall well-being of the offspring.

### 4.5. On Ovarian Cyclicity, Reproductive Capacity, and the Estrous Cycle

To elucidate the preserved reproductive capacity despite these ovarian alterations, it is critical to first consider the factors and parameters affecting fertility (reproductive capacity) in this context. Our results indicate that PM2.5 exposure, particularly from wood smoke, significantly disrupted ovarian cyclicity—evidenced by longer estrous cycles and reduced numbers of antral and growing follicles (Table 6)—and may have impaired follicular development through mechanisms such as endocrine disruption, oxidative stress, and the activation of aryl hydrocarbon receptors (AHRs), as supported by previous studies [67,68]. These disruptions, however, did not appear to directly compromise fertility parameters like the mating rate, fertility rate, or pregnancy rate, which remained comparable between groups (e.g., fertility rate of 91% in FA vs. 83% in NFA, *p* = 0.540; Table 6). This suggests that the observed effects on ovarian cyclicity are more closely related to alterations in follicular dynamics and ovulatory function, which may indirectly influence fetal development—such as reduced litter size and implantation rates—rather than directly impacting fertility itself. The preservation of reproductive capacity despite these disruptions points to potential compensatory mechanisms. These could include the increased recruitment of primordial follicles, enhanced hormonal regulation (e.g., elevated FSH or LH levels to stimulate follicular development), or adaptive changes in the hypothalamic–pituitary–gonadal axis to maintain ovulation and mating success under stress. However, these mechanisms remain speculative and require further investigation to confirm their existence and effectiveness, particularly in the context of chronic PM2.5 exposure. While our findings suggest a dissociation between ovarian cyclicity and fertility, the observed impacts on fetal parameters (e.g., litter size, implantation rates) indicate that PM2.5 exposure may primarily affect gestational outcomes rather than the initial reproductive capacity, highlighting the need for longitudinal studies to fully disentangle these relationships. This study demonstrates a relationship between PM2.5 exposure and alterations in ovarian cyclicity and follicular morphology, evidenced by a lower frequency of estrous cycles and an increase in cycle duration and estrus phase length. A significant reduction in the number of antral and growing follicles—critical for ovulation and estrogen production—was observed. These findings align with previous studies linking air pollutant exposure, including PM2.5, to adverse effects on fertility, such as disruptions in estrous cyclicity and follicular development [25,68]. The literature suggests that air pollutants affect the estrous cycle through mechanisms involving endocrine disruptors, oxidative stress, and epigenetic changes that impair fertility [67,68,69,70]. These mechanisms may disrupt ovarian function by activating aryl hydrocarbon receptors (AHRs) and interfering with the hypothalamic–pituitary–gonadal axis, thereby affecting menstrual and reproductive parameters [71]. The dissociation between disrupted ovarian cyclicity and maintained reproductive capacity could result from compensatory mechanisms or alternative pathways that sustain fertility under adverse conditions. These findings highlight the need to explore additional indicators of ovarian health and reproductive function, as the evaluated parameters may not fully capture the more subtle effects of wood smoke exposure. Further research is necessary to uncover the underlying mechanisms and their implications for long-term reproductive health.

### 4.6. Limitations

This study provides valuable insights into the effects of environmental pollution on female reproductive health using an experimental animal model, highlighting its relevance in understanding potential biological mechanisms. However, certain limitations must be acknowledged. Although the inorganic composition of PM2.5 was characterized, the specific components responsible for the observed reproductive effects remain unidentified, necessitating further research on both organic and inorganic constituents to clarify their toxicity. Another critical limitation concerns the uncertainty regarding whether PM2.5 exerts direct effects on ovarian tissue or acts through indirect mechanisms, as visualizing particle deposition remains methodologically challenging due to their heterogeneous distribution in biological systems [72]. Additionally, the parameters assessed in this study may not encompass the full scope of pollution-induced reproductive effects, emphasizing the necessity of future investigations into molecular and pathophysiological mechanisms. Additionally, significant alterations in ovarian cyclicity and follicular composition—evidenced by longer estrous cycles (23.7 ± 1.23 days in NFA vs. 20.7 ± 1.97 days in FA; *p* = 0.0001) and reduced follicular counts (e.g., fewer antral follicles in NFA, 87.4 ± 32.5 vs. 120 ± 25.7 in FA; *p* = 0.0020) as shown in Table 6—were observed, while overall reproductive capacity, including mating, fertility, and pregnancy rates, remained comparable between groups (e.g., fertility rate of 91% in FA vs. 83% in NFA; *p* = 0.540). In this study, ‘ovarian structure’ refers to the histological organization and quantification of ovarian follicles, assessed through stereological methods, reflecting the presence and distribution of follicular populations (small, growing, antral, and corpus luteum). However, the observed persistence of reproductive capacity despite these alterations suggests the potential existence of compensatory mechanisms, such as increased follicular recruitment or hormonal adjustments, that may mitigate the adverse effects of PM2.5 exposure on ovarian function. These mechanisms, however, remain speculative and require further investigation to confirm their nature and effectiveness, particularly given the ambiguity of distinguishing between ovarian structure and function, as the size and number of follicles may also reflect ovarian age or function rather than solely structural changes. This limitation highlights the need for more detailed histological and functional analyses in future studies to better understand the impacts of PM2.5 on reproductive health. Furthermore, a critical limitation of this study is its reliance on rats as an animal model, which, while valuable for understanding the reproductive effects of PM2.5 exposure, may not fully reflect human physiology and anatomy, particularly in reproductive biology. Although rats share certain biological mechanisms with humans—such as pathways of oxidative stress and endocrine disruption induced by air pollution—significant differences exist, including in ovarian cycles and hormonal regulation. Therefore, while our findings provide foundational insights into the potential impacts of wood smoke pollution on reproductive health, observational studies in human populations are essential to determine whether the same effects occur and to validate the translational relevance of our results for human health protection. Lastly, it is important to highlight that in environmental toxicology studies, considering the potential influence of confounding factors is essential, as these can modulate the magnitude and direction of the observed effects. In the present study, which focuses on evaluating the impact of maternal exposure to PM2.5 from wood combustion on reproductive health, we acknowledge that variables such as environmental stress, diet, and genetic variability could influence reproductive outcomes. Such studies will contribute to a more comprehensive understanding of the consequences of wood smoke pollution on reproductive health. In line with these gaps, future studies should focus on the specific identification of the organic components of PM2.5 and their contribution to the observed reproductive effects. Additionally, integrating molecular biology analyses will allow for the evaluation of alterations in cellular signaling pathways, oxidative damage, and endocrine disruption mechanisms at the ovarian and placental levels. Epidemiological studies could further complement these findings by establishing associations between maternal exposure to PM2.5 and its reproductive effects in human populations. This combination of approaches will provide a more comprehensive understanding of the consequences of wood smoke pollution on reproductive health and contribute to the development of more effective mitigation strategies.

## 5. Conclusions

Finally, this study assessed the impact of PM2.5 exposure on reproductive health, highlighting a decrease in litter size and the implantation rate, confirming the negative influence of air pollution on fertility and fetal development. Alterations in estrous cyclicity and a reduction in ovarian antral follicles were observed, affecting both fertility and mating time. The adverse effects were more pronounced with early exposure to unfiltered air, emphasizing the importance of protecting vulnerable populations from atmospheric pollution and underscoring its critical impact on fetal birth weight and post-implantation loss rate. This study highlights the impact of PM2.5 from wood combustion on reproductive health, emphasizing the need for stronger regulations and mitigation strategies to reduce population exposure. Public policy plays a crucial role in preventing adverse effects on fertility and fetal development, particularly in regions where wood is a primary household fuel. Key interventions include stricter air quality policies, programs to replace wood heaters with cleaner technologies, incentives for sustainable biofuels, and public education on the risks of chronic exposure to fine particulate matter. Additionally, environmental monitoring systems in high-pollution areas could support health alerts and preventive protocols for vulnerable groups, such as pregnant women. While this study focuses on the reproductive effects of PM2.5, its findings underscore the urgency of multidisciplinary strategies that integrate scientific research, environmental regulation, and community education to mitigate air pollution and protect public health. In conclusion, exposure to PM2.5, particularly from wood combustion, significantly affects estrous cyclicity and follicular morphology, suggesting an alteration in ovarian function without a notable influence on overall reproductive capacity. Despite the evident hormonal and ovulatory alterations, compensatory mechanisms within the reproductive system or the activation of alternative pathways appear to preserve fecundity. Nevertheless, the adverse effects on fetal weight and the rate of post-implantation loss demonstrate a clear negative impact on fetal development. These results highlight the need for mitigation policies and air pollution control to protect reproductive health, as well as the importance of expanded research to fully understand the underlying mechanisms affecting fertility and gestational development in response to air pollutant exposure.

## Figures and Tables

**Table 1:** Average concentrations of particulate matter (PM) and carbon monoxide (CO) during the study period and annually. The table presents the mean concentrations of PM2.5, MP10, and CO recorded during the study period (15 June–30 September 2021) and annually. Values are expressed as the mean ± standard deviation (SD), with the coefficient of variation (CV, %) indicating data variability. Pollutant levels were higher during the study period compared to annual averages, particularly for PM2.5 (48.8 vs. 26.2 µg/m^3^) and MP10 (56.9 vs. 36.6 µg/m^3^), likely due to increased emissions from residential wood combustion in winter. CO also showed seasonal elevation (0.78 vs. 0.44 ppm). The highest recorded concentration was for MP10 during the study period (56.9 µg/m^3^), while CO exhibited the greatest annual variability (CV = 97.7%), suggesting significant fluctuations throughout the year.

	Study Period	Annually
PM2.5 [ug/m^3^]	48.8 ± 36.1 (CV: 74.0%)	26.2 ± 30.6 (CV: 117%)
MP10 [ug/m^3^]	56.9 ± 38.3 (CV: 67.3%)	36.6 ± 30.8 (CV: 84.2%)
CO [ppm]	0.78 ± 0.49 (CV: 61.5%)	0.44 ±0.43 (CV: 97.7%)

**Table 2:** Elemental composition of PM2.5 Determined by SEM-EDX. The table presents the relative elemental composition (%) of PM2.5, analyzed using scanning electron microscopy with energy-dispersive X-ray spectroscopy (SEM-EDX). The values indicate the mean percentage of each detected element, along with the standard deviation (SD) and minimum and maximum concentrations observed. Oxygen (44.03%) and carbon (24.00%) were the most abundant elements, followed by silicon (14.15%), likely reflecting mineral and organic contributions to airborne particulate matter. Other detected elements, such as aluminum (5.25%), iron (3.25%), and sodium (2.50%), suggest potential sources from soil, combustion, and anthropogenic activities.

	Mean	SD	Min.	Max.
O	44.03	6.89	39.08	54.23
C	24.00	7.35	16.93	32.06
Si	14.15	7.89	5.51	24.16
Al	5.25	2.97	1.44	7.62
Na	2.50	1.31	0.92	4.11
Cl	1.95	1.50	0.60	4.07
Fe	3.25	1.76	0.90	5.15
Zn	1.60	1.00	0.57	2.67
Ca	0.83	0.75	0.00	1.81
K	1.32	1.00	0.00	2.43
Mg	0.44	0.53	0.00	1.04
S	0.26	0.27	0.04	0.66
Ti	0.32	0.36	0.00	0.64
Pb	0.21	0.31	0.00	0.67

**Table 3:** Principal component analysis (PCA) after varimax rotation for the elements analyzed in PM2.5.

Rotated Component Matrix		
	PC	
	1	2
O	−0.614	−0.743
C	0.959	0.085
Si	−0.883	−0.431
Al	0.029	0.773
Na	0.799	0.583
Cl	0.852	0.389
Fe	0.073	0.962
Zn	0.515	0.739
Ca	0.826	0.519
K	0.076	0.977
Mg	0.756	−0.065
S	0.950	−0.090
Ti	0.285	0.798
Pb	−0.656	0.730

Extraction method: principal component analysis. Rotation method: varimax with Kaiser normalization. The rotation converged in 3 iterations.

**Table 4:** Fixed-effects analysis of a mixed linear model evaluating the difference in weight between groups.

	Estimate	Std. Error	*t*-Value	Pr(>|t|)
FA/FA	279.393	9.299	30.046	6.07 × 10^−10^ ***
FA/NFA	−23.615	2.772	−8.519	3.35 × 10^−14^ ***
NFA/FA	−15.143	2.906	−5.211	7.13 × 10^−7^ ***
NFA/NFA	−6.714	2.762	−2.431	0.0164 *

The intercept represents the average weight of the reference group (FA/FA). The coefficients for the FA/NFA, NFA/AF, and NFA/NFA groups show differences in weight compared to the reference group, adjusting for variations over time. ***: Extremely significant (*p* < 0.001); *: Statistically significant (0.01 ≤ *p* < 0.05).

**Table 5:** A comparison of weight contrasts between groups in a mixed linear model using the Kenward–Roger method for degrees of freedom and Tukey’s *p*-value adjustment for multiple comparisons.

	Estimate	Std. Error	df	*t* Ratio	*p*-Value
FA/FA—FA/NFA	23.61	2.77	131	8.519	<0.0001
FA/FA—NFA/FA	15.14	2.91	131	5.211	<0.0001
FA/FA—NFA/NFA	6.71	2.76	131	2.431	0.0764
FA/NFA—NFA/FA	−8.47	2.72	130	−3.117	0.0119
FA/NFA—NFA/NFA	−16.9	2.56	130	−6.595	<0.0001
NFA/FA—NFA/NFA	−8.43	2.71	130	−3.112	0.0121

The differences between the groups are presented with their respective estimates, standard errors, *t*-ratios, and *p*-values. A low *p*-value indicates a statistically significant difference between the compared groups, with the Tukey method ensuring accuracy when evaluating multiple contrasts.

**Table 6:** Ovarian cyclicity, reproductive capacity, and estrous cycle parameters.

		FA			NFA			*p*-Value
		n	Mean (SD)	CV (%)	n	Mean (SD)	CV (%)	
Ovary cyclicity	N° of cycles per study period	12	2.0 (0.6)	30.2	12	1.42 (0.5)	36.3%	0.0432
	Estrous cycle length (days)	12	20.7 (1.97)	9.53%	12	23.7 (1.23)	5.2%	0.0001
	Estrus phase length (days)	12	4.0 (0.70)	17.7%	12	5 (0.76)	15.4%	0.0057
	Days of estrus/cycle (%)	12	19.5 (3.92)	20.1%	12	21.2 (3.4)	16.1	0.1724
Follicles	Small	6	314 (96.8)	30.9	6	333 (83.8)	25.1	0.5176
	Growing	6	144 (81.3)	56.7	6	97.4 (33.9)	33.9	0.0317
	Antral	6	120 (25.7)	21.4	6	87.4 (32.5)	31.7	0.0020
	Corpus luteum	6	93.8 (36.4)	38.8	6	79.8 (28.3)	35.5	0.2048
Reproductive capacity	Mating rate (%)	12	100		12	100		ns
	Mating time (days)	12	4.67 (2.27)	48.6%	12	3.58 (1.51)	42.0%	0.1456
	Fertility rate (%)	12	91.7		12	83		0.540
	Pregnancy rate (%)	12	100		12	100		ns

**Table 7:** Gestational outcomes in the four studied groups (mean ± SD).

	FA/FA	FA/NFA	NFA/FA	NFA/NFA	*p*-Value
N° of live fetuses per litter	13.5 (2.17)	11.2 (3.70)	10.2 (1.92)	7.8 (1.79)	0.0125
N° of dead fetuses per litter	0 (0)	0.6 (0.89)	0.2 (0.44)	0.4 (0.54)	0.3485
implantation rate (%)	14 (2.37)	11.8 (4.21)	8.4 (3.44)	8.2 (1.64)	0.0149
pre-implantation loss (%)	4.33 (3.39)	6 (6.04)	4.2 (6.02)	13 (9.41)	0.1323
post-implantation loss (%)	0 (0)	4.2 (6.02)	1.6 (3.58)	5.4 (7.80)	0.3203

## Data Availability

The datasets generated and analyzed during this study are available from the corresponding author upon reasonable request.
